# Post-splenectomy native valve endocarditis caused by coagulase negative staphylococci: A rare case report

**DOI:** 10.1016/j.amsu.2022.103929

**Published:** 2022-06-03

**Authors:** Ishak Ahmed Abdi, Abdirahim Ali Adan Nur, Mesut Karataş, Mohamed Farah Yusuf Mohamud

**Affiliations:** Mogadishu Somali Turkish Training and Research Hospital, Mogadishu, Somalia

**Keywords:** Native valve, Endocarditis, Coagulase negative staphylococci, Post splenectomy

## Abstract

Native valve endocarditis caused by coagulase negative staphylococci (CoNS) were a seldom condition, nevertheless there is an increasing CoNS native valve involvement. Here we present a 43 year old patient who underwent Splenectomy due to abdominal trauma he suffered a month before who encountered mitral valve endocarditis caused by coagulase negative staphylococci.

## Highlights

• The various etiologies of cardiac masses in Africa are different than those in higher income settings.
• Native valve endocarditis caused by coagulase negative staphylococci (CoNS) were a seldom condition, nevertheless there is an increasing CoNS native valve involvement.
• Here we emphasize that post spleenectomy can be a risk factor for native valve infective endocarditis caused by non biofilm related Coagulase negative Stephalococus.

## 1 Introduction

The spleen is the body's largest lymphoid organ and serves as a mechanical filter for antigens and microbes. The spleen's capacity to eliminate microorganisms that are encapsulated is particularly important. As a result, patients with asplenia and hyposplenia are more vulnerable to fulminant infections [post-splenectomy infections (PSIs)] [1].

Infectious endocarditis is characterized by infection of a native or prosthetic heart valve, the endocardial surface, or a cardiac device implanted in the patient [2]. Despite its rarity, with an annual incidence of 2–12 instances per 100,000 individuals, infectious endocarditis is a life-threatening condition that causes significant morbidity and disability [2,3].

Although coagulase-negative staphylococcus (CNS) is commonly thought of as a contaminant, reports of particular strains causing endocarditis are becoming more common [4].

We present a 43 year old patient who underwent splenectomy due to trauma before a month who developed native mitral valve endocarditis caused by coagulase negative staphylococci.

## 2 Case presentation

43 years old male presented with weakness, high grade fever and night sweat to the emergency department. He denied any cough, shortness of breath, chest pain, palpitation or hemoptysis. The rest of systemic reviews were unremarkable. He denied having any chronic disease or taking any chronic medication or illicit drug use, his history was significant for Splenectomy before a month ago after a car accident. He is active smoker and works as an army. He appeared to be chronically ill and was not experiencing acute distress. At the time of the presentation his vitals were notable of temperature 38.6C, heart rate of 100/min, Blood pressure of 120/80, Respiratory rate of 14/min, and Oxygen saturation of 98% on room air.

On physical examinations the patient appeared as a cachexic, there were no visible dental lesions in the oral cavity and no cutaneous signs of infective endocarditis, he was pallor and had hyperdynamic precordium, rumbling pansystolic murmur radiating to the armpit grade IV/VI, while the other physical examinations were unremarkable.

The patient's investigation showed a marked leukocytosis (WBC 25.8 × 1000/mm^**3**^ with 83% neutrophils, CRP 174 mg/dl). HIV, hepatitis B virus and hepatitis C virus was negative.

Chest X-ray was clear, urine analysis showed no abnormalities and ECG showed sinus tachycardia and was negative for any dynamic ST changes.

**Fig. 1:**
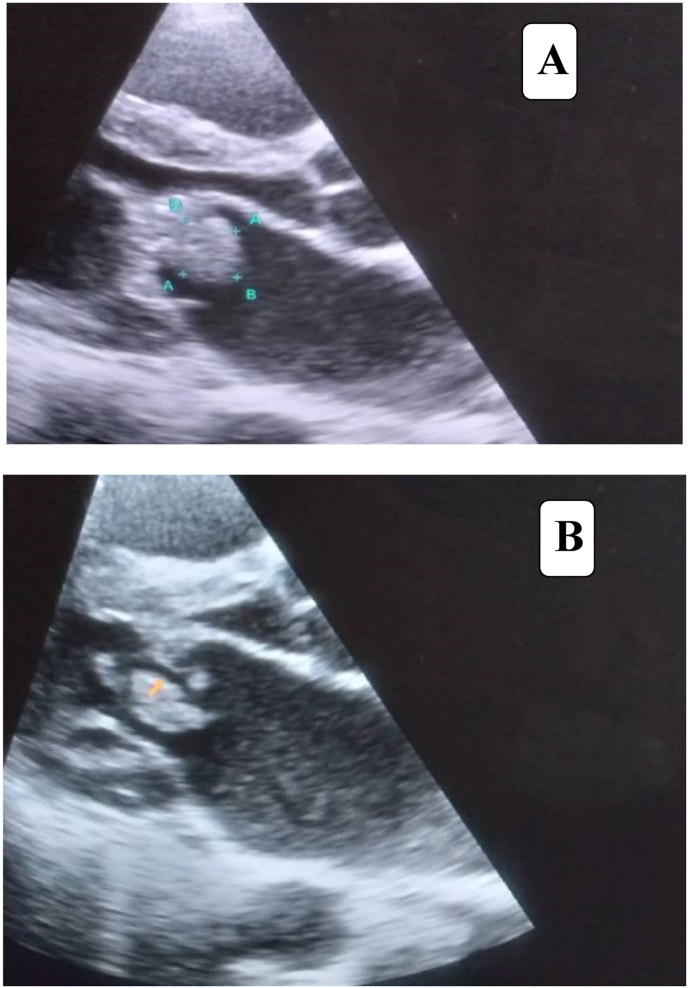
**(A&B)**: Transthoratic echocardiography shows an oscillating mass 16mm*6mm attached to the anterior of mitral valve (typical of a vegetation) and letf atrium dilatation 42MM (A). Typical destructive vegetation with clear prolapse of the mass in to left atrium (B).

**Fig. 2:**
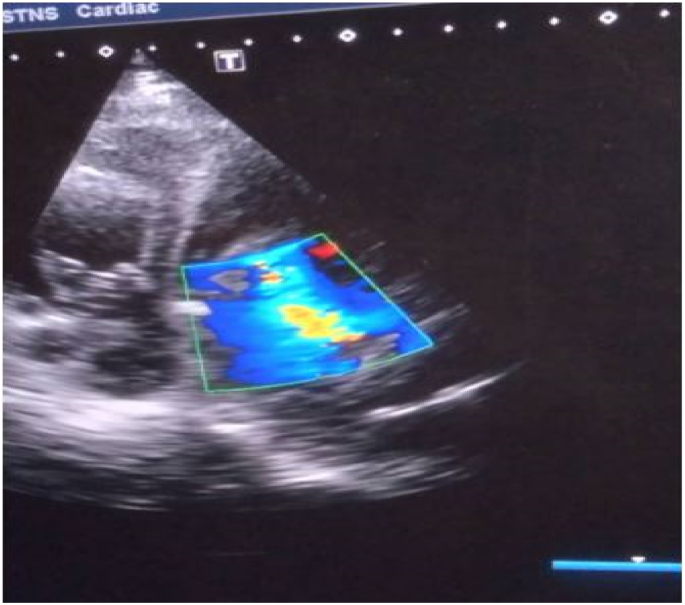
Transthoratic echocardiography shows moderate mitral regurgitation with posterior eccentric jet.

A Transthoracic echocardiography showed ejection fraction of 60% and no regional wall motion abnormalities were detected. There was oscillating mobile mass of 16mm*6mm over the anterior mitral valve; typical destructive vegetation with clear prolapse of the mass in to left atrium associated with moderate mitral regurgitation with posterior eccentric jet and dilated left atrium (Fig. 1, Fig. 2).

Four sets of blood culture were drawn and intravenous vancomycin (1g twice per day), gentamicin (80mg twice per day), and meropenem (3g three times perday) were started while pending blood culture.

**Fig. 3:**
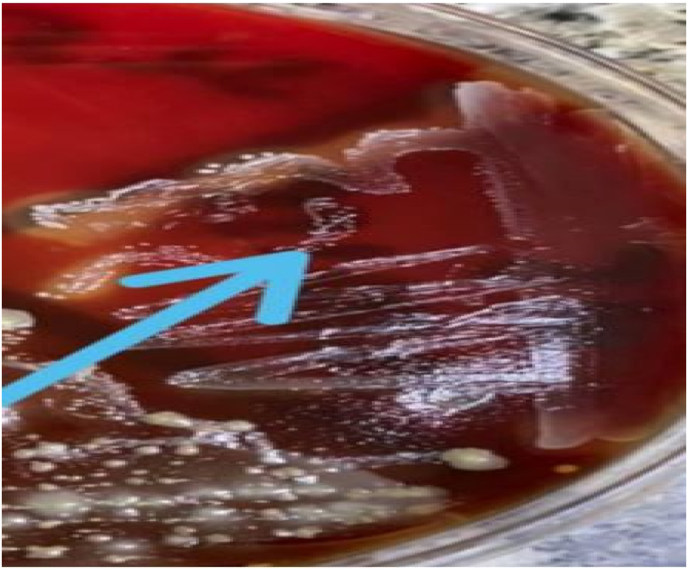
Blood ager media Shows Gram + ve coci.

After four days the gram stain revealed positive for gram-positive cocci in clusters (Fig. 3). The culture was positive of Coagulase Negative Staphylococci in all four culture bottles. Susceptibility testing showed resistance to Ampicilin, erythromycin, and trimethoprim-sulfamethoxazole as shown in Table 1.

**Table 1:** Summarizes the antibiotic susceptibility of coagulase negative Staphelococus organism.

Antibiotics	Susceptibility
Ampicillin	Resistant
Clindamycin	Sensitive
Trimethoprim-sulfamethoxazole	Resistant
Cefoxitin	Sensitive
Erythromycin	Resistant
Linezolid	Sensitive
Vancomycin	Sensitive
ciprofloxacin	Sensitive

Meropenem were stopped after culture result revealed gram + ve coci. Intravenous vancomycin (1g twice per day) and gentamicin (80mg twice per day) was continuing because of their gram + ve synergism and renal function test was performed every two days.

After 2 weeks of antibiotic therapy, there was clinical improvement and inflammatory markers came down. Eventual resolution of the infected state occurred, while TTE revealed a reduction in the size of the mitral valve vegetation and the patient was discharged in a good health with clindomycine 300mg three times per day for four weeks.

He was monitored in follow-up at 4 weeks with asymptomatic and resolution of the mitral valve vegetation.

## 3 Discussion

Coagulase-negative staphylococci (CoNS) are Gram-positive cocci that are immobile, non-spore producing, catalase-positive, usually facultative anaerobes, and lack the enzyme coagulase. They are Gram-positive cocci that are presented as single cells or with irregular disposition. CoNS are bacteria that are found in the human microbiome, which are opportunistic pathogens that cause infections in immune-compromised and prosthetic patients [5]. CoNS was once thought to be a less dangerous pathogen that only caused opportunistic infections or infections of foreign materials like as in situ grafts and prosthetic valve endocarditis. This historical perspective is shifting; however, considerable increases in the prevalence of CoNS-induced native valve endocarditis have been recorded in recent years [4]. In native valve endocarditis, the prevalence of CoNS is considered low. The most common percentages given are 1–3%. However, in some populations, such as a large cohort of 2212 individuals with infective endocarditis a higher prevalence was seen [6].

Our case report demonstrates a native valve coagulase negative endocarditis in post-Splenectomy patient. CoNS were repeatedly and exclusively isolated on serial blood cultures.

Six weeks of vancomycin and rifampin therapy for CoNS prosthetic valve endocarditis was recommended by The American Heart Association and Infectious Diseases Society of America [7].

The organism was isolated in three different blood cultures of two sets of blood culture taken in 24 hours apart. After several regimen of vancomycin and rifampin, the patient didn't need surgical intervention and the inflammatory markers come down with regression of the vegetation.

The main limitation of our case is that further sub classification biochemical tests of CoNS aren't available in our center.

In conclusion, we presented a post-Splenectomy case of a 43 years old man with mitral valve endocarditis caused by CoNS.Our findings highlight the rarity of CoNS endocarditis, with just a few cases recorded, particularly in post Splenectomy and immunocompromised patients.

## Data sharing statement

All original data are available in the Mogadishu Somali Turkish Training and Research Hospital, Mogadishu, Somalia. Data used to support the findings of this study are available from the corresponding author upon request.

## Ethics approval

Based on the regulations, our institutional review board was not required ethic committee approval for case reports.

## Registration of research studies

Name of the registry: Not Applicable.

Unique Identifying number or registration ID: Not Applicable.

Hyperlink to your specific registration (must be publicly accessible and will be checked): **Not Applicable**.

## Guarantor

As Corresponding Author, I confirm that the manuscript has been read and approved by all named authors.

## Consent for publication

Written informed consent was obtained from the patient for publication of this case report and accompanying images. A copy of the written consent is available for review by the Editor-in-Chief of this journal on request.

## Author contributions

Both authors performed substantial contributions to accession of data, or analysis, conception and design, and interpretation of data. Took part in drafting the article or revising it critically for important intellectual content and gave final approval of the version to be published.

## Funding resources

We declared that we have not received any financial support.

## Disclosure

We declared that we have no conflicts of interest.

## Declaration of competing interest

We declare that we have no conflict/competing interests.
